# A Bayesian hierarchical latent trait model for estimating rater bias and reliability in large-scale performance assessment

**DOI:** 10.1371/journal.pone.0195297

**Published:** 2018-04-03

**Authors:** Kaja Zupanc, Erik Štrumbelj

**Affiliations:** Faculty of Computer and Information Science, University of Ljubljana, Ljubljana, Slovenia; Universita degli Studi del Piemonte Orientale Amedeo Avogadro, ITALY

## Abstract

We propose a novel approach to modelling rater effects in scoring-based assessment. The approach is based on a Bayesian hierarchical model and simulations from the posterior distribution. We apply it to large-scale essay assessment data over a period of 5 years. Empirical results suggest that the model provides a good fit for both the total scores and when applied to individual rubrics. We estimate the median impact of rater effects on the final grade to be ± 2 points on a 50 point scale, while 10% of essays would receive a score at least ± 5 different from their actual quality. Most of the impact is due to rater unreliability, not rater bias.

## Introduction

Performance assessment plays a fundamental role in society, especially in education, where it is common practice to test students and base their assessment on test scores. Decisions that follow from performance assessment often have profound consequences for those involved, such as allocation of funding, promotion, and, as is the case with the essay scoring data used in this study, enrolment into higher-educational programs.

Some skills can be assessed with relatively structured tasks, but writing skills and language proficiency are typically assessed with essays, unstructured essay-type questions, or other types of free-response tasks. Scoring these types of performance assessments relies heavily on human judgement. That makes the scores subjective and vulnerable to rater effects. That is, scores can be affected by factors not related to the ability being assessed, such as bias (strictness, leniency) and (un)reliability (non-systematic error) of the rater.

Methods for dealing with rater effects include adding more raters, rater training, score negotiation, the use of scoring rubrics, and rater bias correction. Making valid inferences from essay scores and managing rater effects therefore first requires a good understanding of rater effects, which is what we focus on in this study. That is, we propose a statistical model for estimating rater effects and apply it to rater scoring data. However, the model can be applied in any setting where a set of performances is rated by a set of raters and where at least some performances are assessed by two or more different raters.

### Related work on rater effects

Most related work is from the domain of essay scoring. This is understandable because essays are a very important and commonly used form of assessment, but at the same time very difficult to score objectively, making it even more important to be able to carefully manage rater effects. While related work is varied in terms of essay topics, target population, and sample size, they are very similar in terms of statistical methodology used. Assessment scores and raters are typically modelled with a mixed linear model, with rater-specific and sometimes time-period, and scoring rubric-specific effects. In most cases, numerical scores are treated as categorical or ordinal variables, and (multivariate) ordinal logistic regression (polytomous [partial credit] Rasch model) is used [1–13]. In some studies, the scores are treated as a continuous random variable and modelled with a mixed linear regression model [14] or a multi-level mixed linear model [15, 16].

The most frequently examined rater effects are rater bias and rater reliability. There is strong empirical evidence in favor of some raters being biased—substantially more severe or lenient [1–3, 5–13, 15–20]. Bias based on writer identity, such as gender or ethnicity bias, have also been reported, but will not be discussed in detail. In large-scale assessment, these can be dealt with using anonymization.

Most studies of free-response assessment also report non-negligible impact of rater bias and (un)reliability on final scores: [18] estimated that 30% of students in the Advanced Placement English Literature and Composition Program would have received a different grade had they accounted for rater reliability (most differences were a grade lower or higher on a 5-grade scale). [15] estimate residual standard deviations of 2.23, 3.16, 3.22 points for team leaders, experienced raters, and new raters, respectively, on a 30-point grading scale. [14] reported a 0.61 residual standard deviation on a 6-point scale for spoken English proficiency scores. [6] showed that 7 out of 10 students in the Golden State Examination writing assessment would have received one point different score when rated by the most severe rater compared to the most lenient rater on a five grade scale. [2] showed that differences between days were 0.14 of a score point, which represented 24% of a standard deviation for a person of average ability in their study. [15] report that the most lenient raters scored, on average, almost 4 points higher than the most severe raters (on a 30-points scale), [16], however, found no practically relevant effects of bias (1.26 on a 100-point grading scale).

Another often researched rater effect are changes in the rater’s bias (severity), reliability, or use of scale categories/rubrics over time (these changes are often jointly referred to as rater drift). Related work analyses drift only over a short period of time (3-7 days), with mixed results. [2] and [13] report changes in bias, [6] report a drift towards the mean (unbiasedness), [15] find no drift, [9] report changes in bias and scoring rubric use. A notable exception in terms study period length is [19], who studied and reported changes in severity over a 3-year period. However, only a small number of raters participated throughout the study and comparisons between years were made between groups of raters not individual raters, so no conclusions can be made regarding long-term intra-rater drift, and [16] report a significant but practically negligible drift in time towards more severe scoring.

Other studied rater effects are: (a) central tendency—there are several studies that provide evidence in favor of raters being biased away from the extremes scores or categories [9, 13, 15, 17], (b) rater experience—[15] found no significant differences in bias or reliability between more and less experienced raters, and (c) category/rubric preferences—[4] found that raters differed significantly in their views on the importance of the various scoring criteria, resulting in several distinct types of raters, [7], [10], and [11] also found consistent differences between raters in terms of scoring criteria importance and [21] found that raters were significantly influenced by mechanical characteristics of students’ writing rather than the content even when they used a scoring rubric.

### Contributions of this study

In most related work, the available number of essays or raters is small (a couple of dozen), which limits model complexity. There are a few studies of large-scale assessments (10.000+ scores) [9, 15, 18], but even these span only a very short period of time (a single essay) and analyse only short term rater drift. Our research is based on large-scale assessment data, which consists of almost 80.000 scores and a stable set of over 200 raters over a period of 5 years and 5 different essays (see Table 1 for details). Each score is broken down into sub-scores according to a standard rubric. These data, described in more detail in the Essay scoring data section, allow us to study several substantive research questions in more detail, compared to related work. First, an accurate estimate of rater bias and reliability. Second, an investigation of long-term rater drift. Third, an analysis of scores by rubric, in particular, the relationship between language (writing mechanics) and content. And fourth, a quantification of the impact of rater effects on final grades.

**Table 1:** Summary of the data broken down by year: Number of essays (n), raters(m), mean score ($\bar{y}$), standard deviation (sd(y)), and median number of scores per rater (*median*(*n*_*r*(*i*)_)).

year	n	m	$\bar{y}$	sd(y)	*median*(*n*_*r*(*i*)_)
2010	9107	167	30.2	7.4	110
2011	8477	185	30.6	7.3	99
2012	7081	177	30.5	7.9	90
2013	7250	172	30.1	6.9	83
2014	6995	153	29.9	7.3	93
all	38910	221	30.3	7.4	381

Related work relies predominantly on null-hypothesis testing for presence of effects, rather than estimating effect size, and, despite a typical large number of parameters (effects), no attempts are made at adjusting for multiple hypothesis tests, which may lead to spurious findings where there are no effects. We develop our statistical model in the Bayesian framework to deal with these issues, simplify interpretation, and facilitate estimation of the impact of rater effects through simulations from the posterior distributions. Our approach is related to the hierarchical rater model (HRM) [22, 23] but with discretized Tobit instead of logit (partial-credit) model for observed scores and an extension of correlated bias and severity across scoring rubrics.

## Essay scoring data

The data used in our research were obtained during the nation-wide external examination conducted by the National Examination Centre in upper secondary schools in Slovenia. This examination, also called Matura, is an obligatory exam, which represents the completion of secondary education and is the main criterion for the enrolment into higher-educational programs. Matura consists of three compulsory (native language—usually Slovenian language, mathematics, and foreign language) and two elective subjects. The basic maximum grade a student can obtain is 28, but this can be raised up to 34 if the student elects to take 2 subjects at a higher level. The essays we analyse in this study are part of the Slovenian language subject exam and represent 50% of the exam score. The total for this exam is 8 so the essay effectively contributes 4 to total of 28 on the Matura.

Matura is held twice per year, spring and autumn, and each term, students can opt for one of two types of essays, argumentative or interpretive. In this study, we only use the spring term argumentative essays for years between 2010 and 2014, inclusive. The reasons behind this choice are that a vast majority of students take the spring exam (autumn exams are typically taken by students who have failed the spring exam, want to improve their score, or were not able to attend the spring exam) and most students opt for the argumentative essay. In the studied period of time, 83% of all essays were spring term argumentative essays. A summary of our data is presented in Table 1.

An essay can receive a score between 0 and the maximum of 50 points. This score is broken down into rubrics: 20 points for the language-related rubrics and 30 for the content-related rubrics. A rater can, at their discretion, assign 6 bonus points, 3 for language and 3 for content, but the sum of all points cannot exceed 50. Points for language are additionally divided into three scoring rubrics: syntax (up to 8 points; grammar, syntax, punctuation, word use, …), style (up to 6 points; loosely defined as measuring the principles of good communication), and structure (up 6 points; title, word count, organization, …). Points for content are also broken down into rubrics, but the number of rubrics and points per rubric vary from year to year, depending on the essay topic. To simplify analysis, we only record and analyse the total of the content related rubrics.

The group of Matura raters consists of upper secondary schools teachers. Raters have to attend trainings and seminars organized by the National Examination Centre and consider detailed criteria when assessing the exams. The group of raters is relatively consistent through the time, since many Matura raters score the matura essays over their entire teaching career. Each essay is scored by two raters and their average is the essay’s final score. In cases where the two scores differ by more than 10 points, a third rater is included, who inspects both scores and his score is the final score.

The data are available for download S1 Data.

## Methods

In the ideal case we would know the quality of each essay, that is, the ground-truth, making it simple to estimate each rater’s bias and variability from how the rater’s scores deviate from and vary around the ground-truth. Note that in the context of the model, we use rater variability instead of rater reliability, but the two are interchangeable.

In some studies, experienced raters’ scores [16] or a scoring committee’s average [15] are used as a proxy for ground-truth. However, this is problematic, as there is no evidence that senior raters are unbiased or have substantially higher reliability (lower variability). Therefore, unless we have a very large committee or a large number of raters per essay, failing to account for variability in such proxy ground-truth will lead to incorrect conclusions, in particular, underestimating the rater’s reliability or falsely concluding that raters exhibit central tendency (avoiding the extremes). Therefore, we cannot assume certainty so it is natural to model an essay’s quality with a latent variable. It is necessary to have scores from 2 or more raters for at least some of the essays, however, because rater and essay quality variability can only be identified from the raters’ mean and variability on the same essay, respectively.

Assessment scores are, by definition, ordered, and, in most cases, numeric and discrete. We can choose to model the scores as continuous or categorical (ordinal) variables. One such suitable model is the ordinal logistic regression model, which is the almost exclusively used approach in related work. We do not base our model on this model, however, for several reasons. The first reason is model complexity—for *k* ordinal categories, *k* − 1 breakpoint variables are required, and, for rater-specific variables, which is necessary if we are to study rater-specific effects, this is multiplied by the number of raters. Furthermore, allowing the breakpoints to vary across raters would complicate the placement of a hierarchical prior distribution and make it more difficult to interpret the bias and variability of the raters.

Instead, we model the observations as a function of a normally distributed latent variable that is discretized to the nearest integer and truncated at 0 and maximum score. Therefore, we keep the number of parameters at a minimum, while allowing for zero- or maximum-inflation, which is relevant for rubrics with a small number of categories. We add essay population and rater population-level distributions and parameters. The hierarchical Bayesian approach serves two purposes. First, the second level provides additional insight into essay and rater properties and simplifies simulation of rater effects using quantities derived from the posterior distributions. And second, shrinkage of essay- and rater-specific parameters towards the population-level mean alleviates the identification of extreme effects purely by chance and the problem of multiple comparisons (see [24] for details).

Therefore, the model in our approach can be viewed as a discretization of the Tobit model [25, 26], truncated at both ends, or as a simplified ordinal probit model with fixed breakpoints between categories and non-unit variance. In the remainder of the section we provide a detailed description of the single score model and its extension to a setting where the total score is broken down into rubrics.

### A model for essay scoring

We are interested in modelling essay scores in a setting where each essay is scored one or more times, each time by a rater from a group of identifiable raters and with a bounded discrete numerical score. Let *N*, *n*, and *m* be the total number of scores, essays, and raters, respectively. We use *w*(*i*) and *r*(*i*) to index the essay and rater of the *i*-th score.

We assume that the true latent quality of an essay *l*_*w*(*i*)_ lies somewhere on an underlying continuum and that the essays perceived quality in the eyes of the rater $l_{w(i)}^{*}$ is the sum of the essay’s true latent quality, the rater’s bias *b*_*r*(*i*)_, and random noise *ϵ*_*i*_:

$$\begin{array}{c} l_{w(i)}^{*}=l_{w(i)}+b_{r(i)}+\epsilon_{i}, \end{array}$$

where the noise is normally distributed with rater-specific variability, *ϵ*_*i*_ ∼ *N*(0, *σ*_*w*(*i*)_)

The actual reported and therefore observed integer score *y*_*i*_ is assumed to be a truncation and discretization of the perceived quality:

$$\begin{array}{c} y_{i}=T\left(l_{w(i)}^{*}\right). \end{array}$$

Function *T* is defined as

$$\begin{array}{c} T\left(x\right)=\left\{ \begin{array}{cc} 0 & \text{if}\;x\leq \frac{1}{2},\\ z & \text{if}\;x\geq z-\frac{1}{2},\\ \text{nint}(x) & \text{otherwise,} \end{array}\right. \end{array}$$

where nint is the nearest-integer function and *z* the maximum number of points for the essay or, if applied to a single rubric, the maximum number of points for that rubric. The minimum number of points is assumed to be 0 always.

We place hierarchical priors on the model’s parameters. The essays latent qualities are assumed to be draws from a normal distribution *l* ∼ *N*(*μ*_*l*_, *σ*_*l*_) and the rater-specific non-systematic errors are assumed to be draws from a normal distribution *σ*_*i*_ ∼ *N*(*μ*_*ϵ*_, *σ*_*ϵ*_) left-truncated at 0.

If the biases *b* are allowed to vary freely, we cannot identify the latent qualities and the biases (an arbitrary constant can be added to either group of parameters and subtracted from the other). To achieve identifiability, we introduce a restricted bias parameter $b_{i}^{*}$, *i* = 1‥(*m* − 1), forcing the biases to sum up to 0: $b_{m}=-\sum b_{i}^{*}$ and $b_{i}=b_{i}^{*}$ for *i* = 1‥(*m* − 1). A hierarchical prior is placed on the biases: b* ∼ *N*(0, *σ*_*b*_). The assumption that raters as a group are expected to be unbiased is natural and it would not make sense to assume that the expectation (consensus) of all raters in a closed world is biased. In theory, this assumption can lead to the same issues as those discussed earlier regarding assuming ground-truth scores. However, in practice the total number of raters and scores per rater are always much higher than the number of different raters per essay.

Finally, priors on the hyper-parameters were set as to allow for the placement of weakly-informative priors: normal priors for the means *μ*_*l*_ ∼ *N*(*ν*_*l*_, *η*_*l*_), *μ*_*ϵ*_ ∼ *N*(*ν*_*ϵ*_, *η*_*ϵ*_) and uniform priors for the standard deviations *σ*_*ϵ*_ ∼ *U*(0, *u*_*ϵ*_), *σ*_*l*_ ∼ *U*(0, *u*_*l*_), *σ*_*b*_ ∼ *U*(0, *u*_*b*_).

#### Measuring rater effects

We estimate the impact of rater effects with simulation from the distribution of the absolute deviation of the average score across *a* raters from the latent score of an essay

$$\begin{array}{c} e\left(a\right)=\left|\frac{1}{a}\sum_{i=1}^{a}T\left(l+b_{i}+\epsilon_{i}\right)-T\left(l\right)\right| \end{array}$$

and, corrected for estimated bias,

$$\begin{array}{c} e_{corr}\left(a\right)=\left|\frac{1}{a}\sum_{i=1}^{a}T\left(l+\epsilon_{i}\right)-T\left(l\right)\right|, \end{array}$$

where *l* ∼ *N*(*μ*_*e*_, *σ*_*e*_), *b* ∼ *N*(0, *σ*_*b*_), *ϵ* ∼ *N*(*μ*_*ϵ*_, *σ*_*ϵ*_), and the parameters are drawn from the posterior distributions.

#### Model variants

We will refer to the above model as the *rater-specific* variability model. In order to investigate how certain parts of the model affect how well the model fits the data, we include two simpler models in the comparison. First, the *common* variability model, which is identical to the rater-specific model, with the exception of random noise variability, which is assumed to be the same for all raters. And second, the *baseline* model, where all the rater effects are omitted (bias and rater-specific variability). For both models, a uniform prior is placed on the common variability.

### Model fitting and evaluation

All statistical analyses were performed using the R programming language [27] and the Stan programming language for (Bayesian) inference [28]. The models were implemented in Stan and fitted using MCMC sampling-based approximation—the No-U-Turn Sampler variant of Hamiltonian Monte Carlo [29]. Sampling was run for a number of iterations sufficient to achieve estimated sampling errors of at least two orders of magnitude lower than the posterior standard deviations and would therefore not have a practical effect on interpretation of the results.

When choosing constants for the hyper-priors, we partially relied on prior knowledge, but only to omit the practically impossible ranges of values for the standard deviation parameters and weakly informative values were placed on the means: *ν*_*l*_ = 30, *ν*_*ϵ*_ = 5, *η*_*l*_ = *η*_*ϵ*_ = 100, and *u*_*ϵ*_ = *u*_*l*_ = *u*_*b*_ = 70. Due to the amount of data available, the posterior distributions were found to be insensitive across a range of more and less restrictive values and practically equivalent to selecting flat priors across the unrestricted ranges for all parameters. However, with restricted ranges, sampling was substantially more effective.

We estimated the models’ goodness-of-fit using approximate leave-one-out cross-validation (LOO-CV) (see [30]) as implemented in the loo package [31]. Note that all reported values of the LOO-CV criterion are transformed to be on the deviance scale. We also performed several visual checks of how the model’s predictions of certain aspects of interest match the observed data.

### Rubrics model

The main purpose of the rubrics model is to estimate the effects of rubric-specific biases and potential correlations between them. A choice was made not to model rater and rubric-specific variability and to model latent quality as independent across rubrics. The main reason was computational convenience and the fact that these would not contribute to bias estimation. Therefore, the rubrics model extends the common variability model, but now the observations are vectors of length *K*, where *K* is the number of rubrics. Similarly, *y*_*i*_, *ϵ*_*i*_, *i* = 1‥*N*, *b*_*i*_, *i* = 1‥*m*, and *l*_*i*_, *i* = 1‥*n* are now also vectors of length *K* and ***y***_*i*_ = *T*_*k*_(***l***_*w*(*i*)_ + ***b***_*r*(*i*)_ + ***ϵ***_*i*_).

For the latent essay qualities and rater variability, we use a similar hierarchical prior structure, independently for each rubric, and similar values for the hyper-prior constants. For the latent qualities $l_{i}∼N_{K}\left(\mu_{l},\sigma_{l}I\right)$, with $\mu_{l}∼N_{K}\left(5,100I\right)$ and $\sigma_{l}∼N_{K}\left(5,100I\right)$ (left-truncated at 0). And for the rater variablity $\epsilon_{i}∼N_{K}\left(0,\sigma I\right)$ and *σ*_*i*_ ∼ *U*(0, 70), for *i* = 1‥*K*. On the biases, however, we put a covariance structure ***b***_*j*_ ∼ *N*_*K*_(**0**, *Σ*_*b*_), decomposing the covariance matrix into scale vector and correlation matrices *Σ*_*b*_ = *AΩA*, where $A=\sigma_{b}I$. Priors are placed separately on the scale *σ*_*b*,*i*_ ∼ *U*(0, 70), for *i* = 1‥*K* and correlation *Ω*∼ LKJ(1). LKJ is the distribution of correlation matrices from [32] and LKJ(1) is equivalent to a flat prior over all correlation matrices.

## Results

### Model comparison and validation

We start with model comparison and visual validation. In Table 2 we compare the three models described in the Methods section. The rater-specific variability model provides the best fit, but does not result in substantially different estimates of bias, compared to the common variability model. Both models that consider bias (common and rater-specific model) fit the data substantially better than the baseline model which does not allow for bias.

**Table 2:** Model evaluation and comparison. Pearson correlation coefficients are included for correlation between the mean posterior bias of a rater and that rater’s mean’s deviation from the grand mean across all raters for current year and the following year, separately.

	baseline	common			rater-specific		
year	LOO-CV ± SE	LOO-CV ± SE	*ρ*	*ρ*_*year*+1_	LOO-CV ± SE	*ρ*	*ρ*_*year*+1_
2010	111382 ± 196	106445 ± 205	0.93	0.40	101385 ± 213	0.93	0.42
2011	104301 ± 184	100136 ± 192	0.92	0.34	96263 ± 195	0.93	0.33
2012	88563 ± 175	84921 ± 175	0.88	0.32	81755 ± 173	0.87	0.31
2013	89128 ± 174	84986 ± 182	0.94	0.42	81679 ± 178	0.94	0.41
2014	86861 ± 167	83322 ± 172	0.88		80750 ± 176	0.85	

Table 2 also shows how the estimated bias correlates with the rater’s deviation from the overall mean score on the current and following year. As we would expect, the correlation is high for the current year, and, although the two are not exactly the same, deviation from the mean would be a decent proxy for estimated bias. However, the standard deviations of scores in Table 1 imply that, even in our case, with several dozen scores per rater, the standard error of such estimates is still relatively high (between 0.5 and 1.0 points).

Correlations of the estimated biases with the next-year’s biases (only for raters who scored both years) suggest that rater bias is partially preserved over time, despite the fact that each year there is a different essay topic. We explore this further in the Rater effects section that follows.

**Fig 1:**
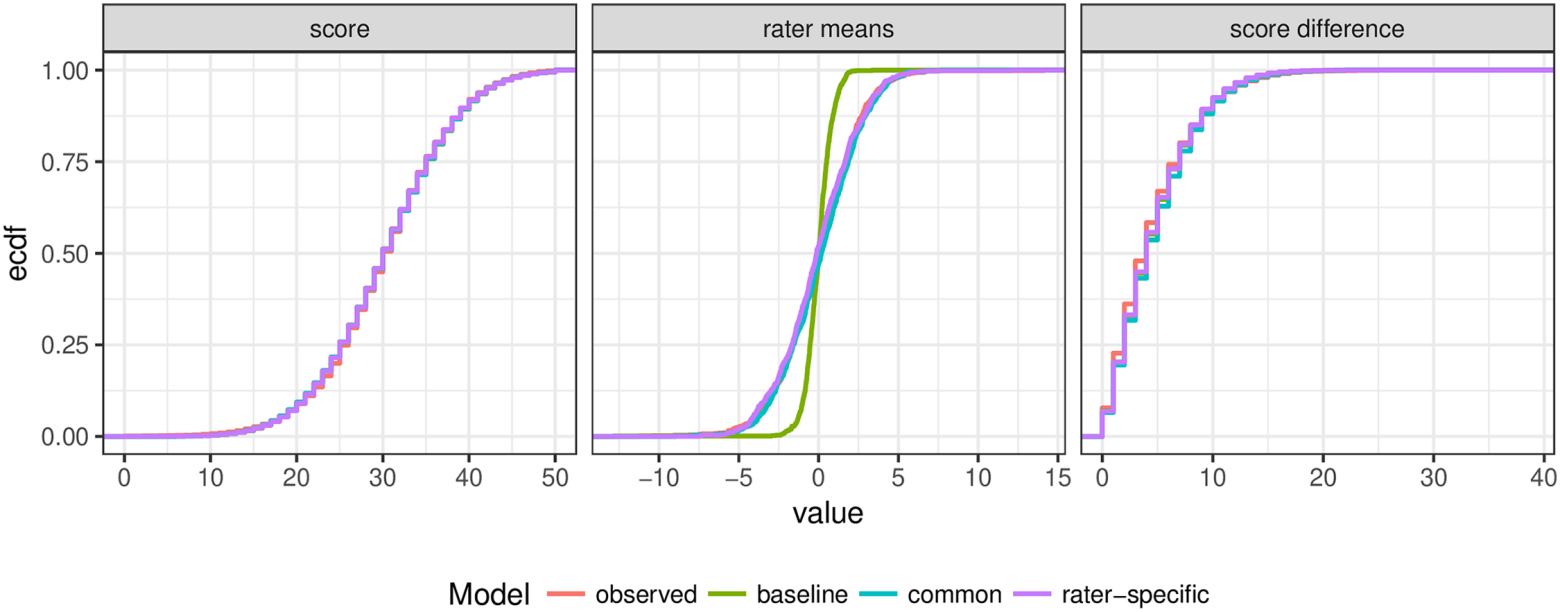
Visual posterior predictive checks. A comparison of the cumulative distributions simulated from each model and the actual observations for three relevant aspects: distribution of essay score, distribution of rater’s mean score (relative to grand mean), and distribution of the absolute difference between the two scores for the same essay.

Finally, Fig 1 visually compares the models in terms of the distributions of some of the quantities of interest replicated from the models and how they match the observed empirical distributions. In all cases, with the exception of the baseline model’s rater means, the distributions match the observed and there are no notable differences between the common and rater-specific variability model. The baseline model matches the distribution of scores, but underestimates the standard deviation of the distribution of rater means around the average score across all essays, which is what we would expect in the presence of rater bias.

### Rater effects

**Fig 2:**
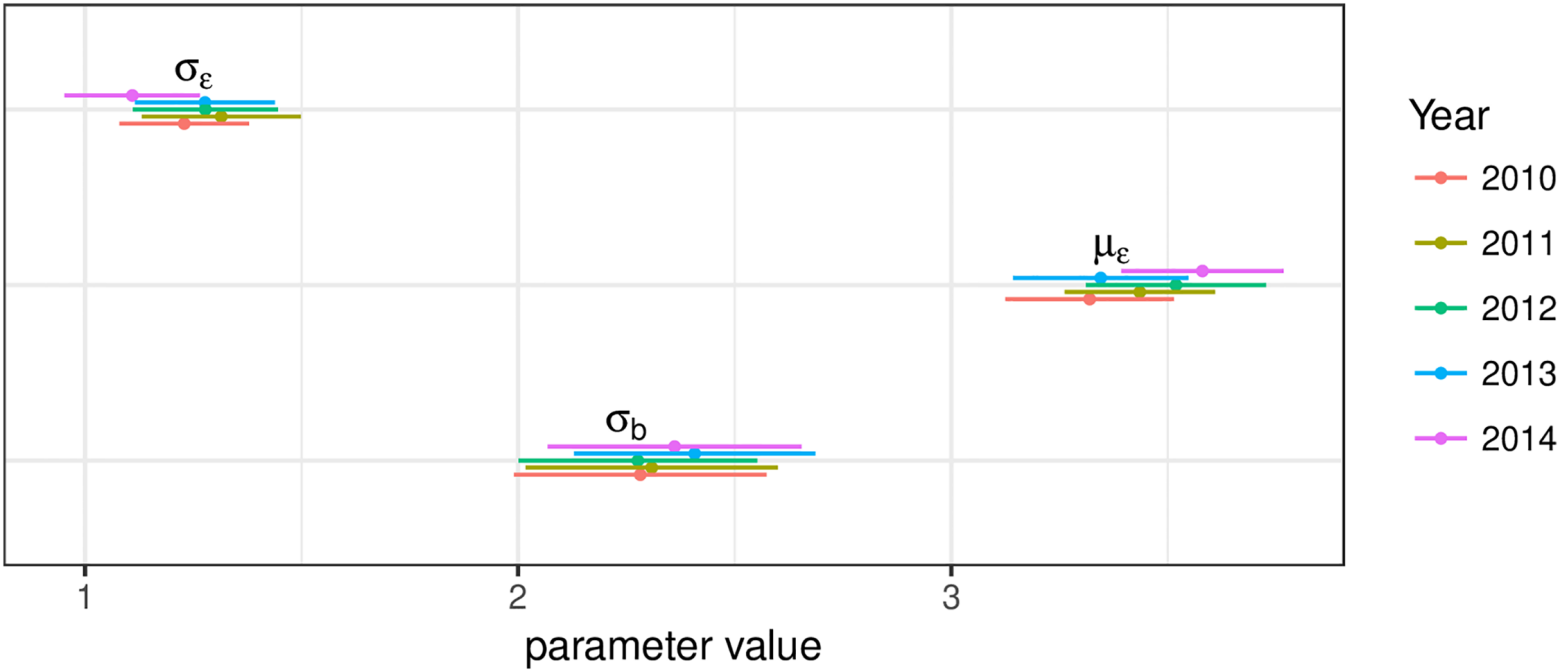
Estimated parameter means and 95%CI.

**Fig 3:**
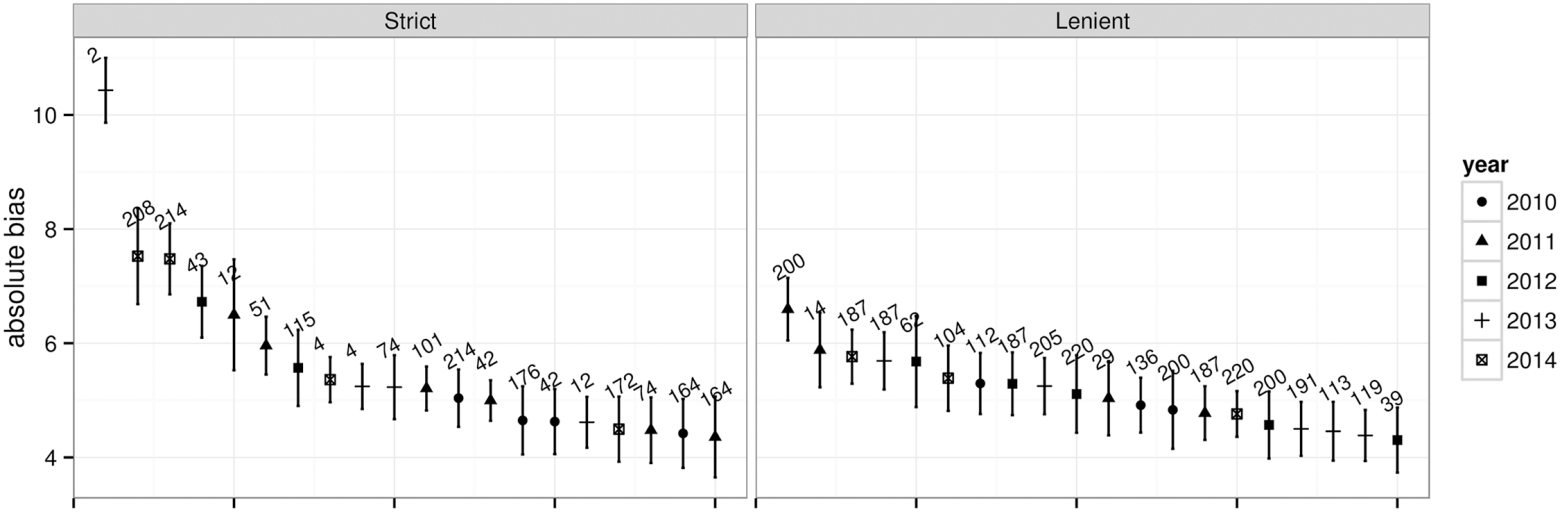
Rater bias. The estimated top 20 most strict and most lenient scoring performances by a rater in a year for all five years. Each point is annotated with the rater’s ID and several raters appear more than once (for performances in different years). The bars indicate the standard deviation of the estimates.

Based on results from the Model comparison section, we used the best model (rater-specific model) to perform all further analyses. Fig 2 shows the bias and variability parameter estimates by year. It can be observed that the simulated rater effects are stable within each year and very similar in all of the studied years, so we opt to pool together the results for rater effects, instead of presenting each year separately. The estimated most biased performances in the observed period of time are shown in Fig 3 and provide a reference for the extreme of the bias and the certainty of the estimates.

Table 3 shows the estimated impact of rater effects on essay scores. In our case, where there are two raters per essay, we estimate that one half of all the scores differ by 2 or more points and 10% of all essay scores differ by 5 or more points from the actual score. By accounting for the bias, the impact is estimated to decrease by 0.5 and 1 point, respectively, but variability/unreliability of rater scores has a substantially stronger impact on grades than bias. Another option that could be considered is adding another rater, which is estimated to have an impact similar to accounting for bias.

**Table 3:** Estimated rater effects for different numbers of raters per essay.

	*e*(*a*)			*e*_*corr*_(*a*)		
raters (a)	median	75%	90%	median	75%	90%
1	3	5	7	2	4	6
2	2	3.5	5	1.5	3	4
3	1.67	2.67	4	1.33	2.33	3.33

Table 4 shows the correlations between estimated posterior bias and reliability between the studied years. We can observe that the correlations are quite high, implying that a rater’s effects partially, but not entirely, persist through the years. This was already indicated in Fig 2 and partially in Table 2, but here we can observe it in more detail and it applies to both bias and variability. Out of the total 221 raters, 95 raters have all mean estimated posterior biases (across all years in which they participated as raters) of the same sign (44 lenient and 51 severe).

**Table 4:** Correlation coefficients between estimated posterior bias and reliability for each pair of the studied years.

	bias					reliability				
years	2010	2011	2012	2013	2014	2010	2011	2012	2013	2014
2010	1	0.55	0.44	0.47	0.53	1	0.5	0.48	0.57	0.46
2011	0.55	1	0.41	0.47	0.51	0.5	1	0.57	0.53	0.5
2012	0.44	0.41	1	0.37	0.42	0.48	0.57	1	0.62	0.49
2013	0.47	0.47	0.37	1	0.47	0.57	0.53	0.62	1	0.64
2014	0.53	0.51	0.42	0.47	1	0.46	0.5	0.49	0.64	1

The between-subject and between-year variability in mean posterior biases account for 60% and less than 1% of the total variability, while the rest is residual variability. For reliability, these numbers are 67% and 1%. These calculations were made assuming fixed bias estimates (taking the posterior means and discarding the uncertainty). If we also take into account the uncertainty in our estimates (the variability of the posterior distributions), it would, in the absolute sense, amount to approximately 25% of the total variability for bias and 30% for reliability. Therefore, it is possible that as little as 15% and 5% of the variability in bias and reliability, respectively, is residual (within-subject variability over time). This uncertainty in our estimates of within-subject variability over time is a consequence of having only 5 points (years) per rater.

**Fig 4:**
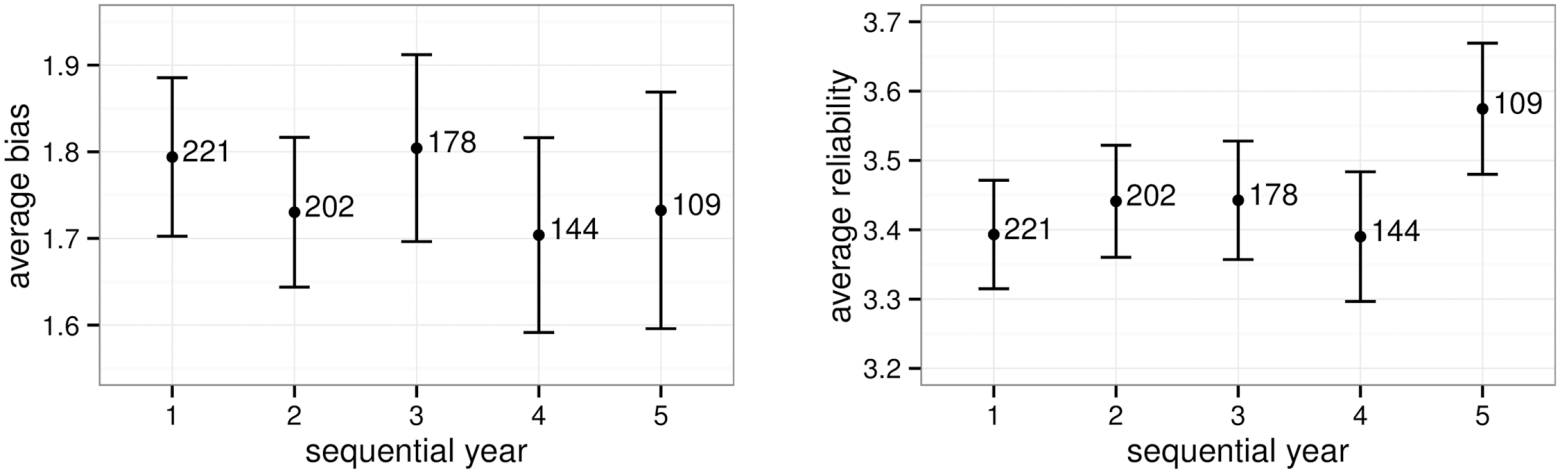
Rater bias and reliability over time. Mean estimated absolute bias and reliability with standard deviations, broken down by year of scoring. For example, year 3 includes the rater/year pairs where it was the rater’s third year of scoring the essays in the observed period. The numbers represent the number of raters—there were 109 raters who scored all 5 years.

We observed in Table 2, Fig 2, and Table 4 that bias and reliability partially persist over time. Another aspect of interest are potential changes of rater effects with rater experience. While rater experience data were not available, we can indirectly and partially infer rater experience from the number of years they participated in scoring. The results are shown in Fig 4 and reveal that there are no discernible drifts in bias or reliability.

### Rubrics

**Fig 5:**
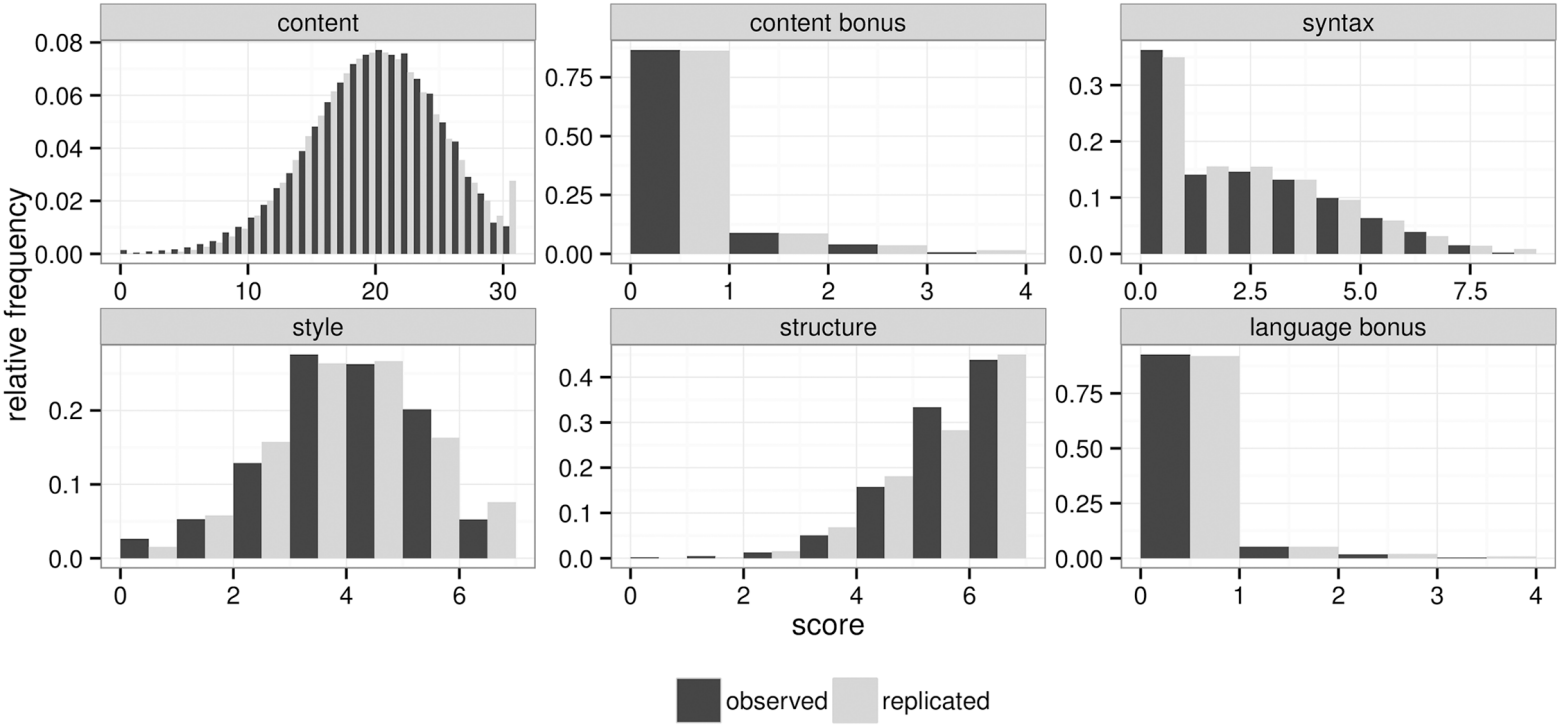
Score distributions. A comparison of the rubrics scores replicated from the rubrics model with the actual observed distributions. Analyses were done for each year separately and pooled together to produce these histograms.

Fig 5 compares the score distributions replicated from the rubrics model with the actual observed distributions. Overall, the proposed model is flexible enough to fit the different distributions, with relatively small maximum score inflation in the content rubric.

Similar to the single total score, we can use the rubrics model to estimate the impact of rater effects for individual rubrics. Table 5 shows that the overall impact of a rubric corresponds to the maximum number of points for that rubric, the content rubric having by far the largest impact. However, after normalization, the style and structure rubrics have the highest impact, followed by content and syntax, and the two bonus rubrics have the least impact.

**Table 5:** The estimated total rater effect (mean absolute difference from ‘true’ score) and relative rater effect (total effect relative to standard deviation of score for rubric).

rubric	effect (points)	rel. effect (%)
content	2.52 ± 0.11	49.51 ± 3.2
content bonus	0.19 ± 0.02	37.09 ± 2.84
syntax	0.95 ± 0.1	49.45 ± 5.59
style	0.95 ± 0.06	69.8 ± 4.58
structure	0.65 ± 0.05	64.11 ± 4.03
language bonus	0.11 ± 0.04	29.64 ± 7.73

**Fig 6:**
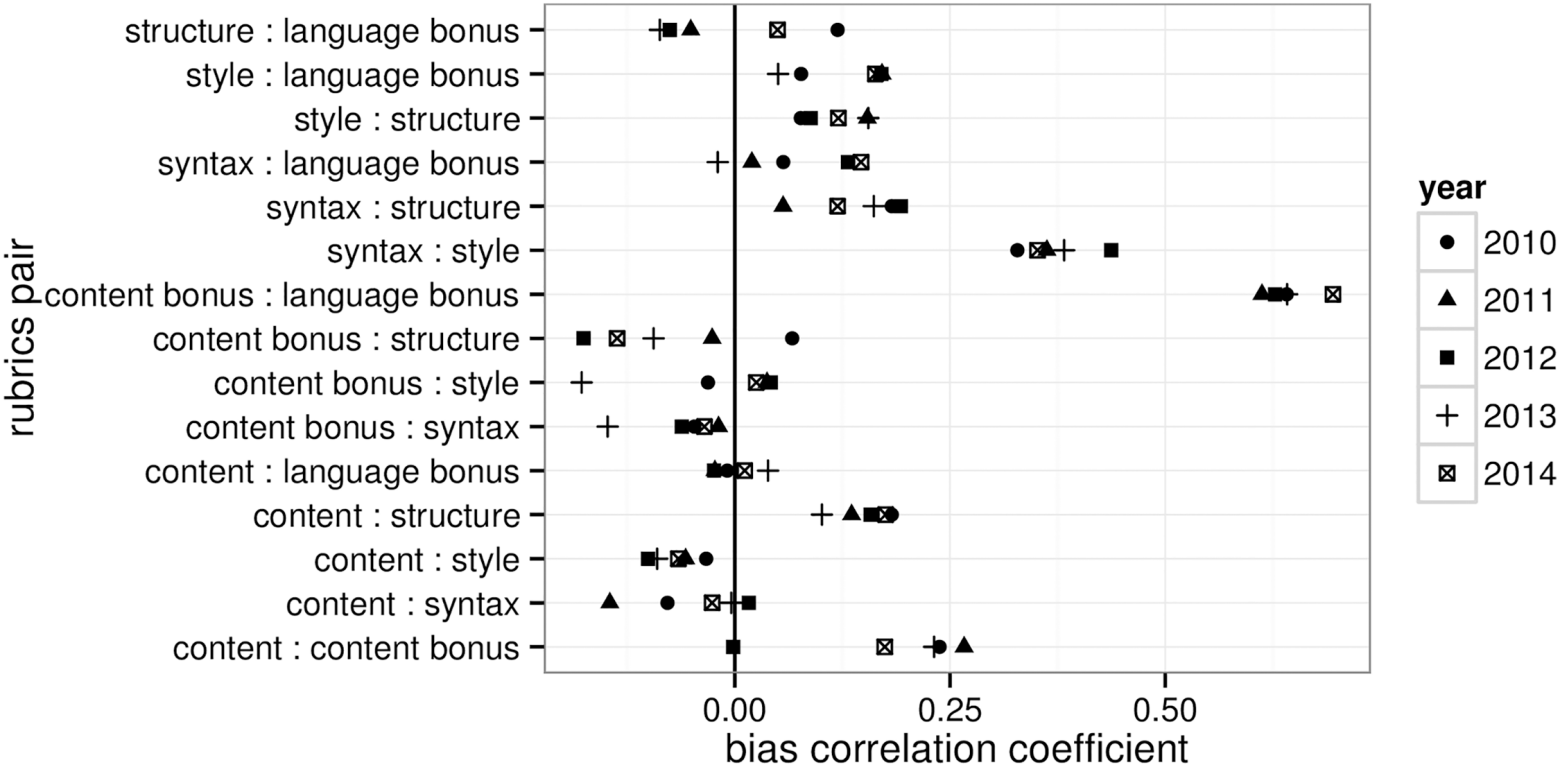
Rubrics correlations. Estimated correlations between biases in different rubrics.

Finally, we investigate if a rater’s rubric specific biases are correlated. That is, do raters biased in one specific rubric tend to be biased in another rubric as well. Fig 6 compares correlation coefficients between estimated bias for every pair of rubrics. The estimated correlations are relatively stable across years and the two bonus points rubrics are by far the most correlated. Positive correlation can also be observed between syntax and style, content and content bonus, and structure and content. Language-related rubric pairs except structure and bonus have non-negative correlation and the content rubric bias has negative correlation or does not correlate with any of the language-related rubrics (except for structure).

## Discussion

The proposed model provides a good fit for both the total score when extended to individual rubrics, despite substantial semantic differences in rubrics and subsequently in their scales and distributions. As a tool, the model can be used to estimate rater effects, their impact on final scores, and to identify the most biased and unreliable raters. The model could also be used to adjust final grades, correcting for bias. However, this is not likely to occur, due to prohibitive legislative issues. While the focus was on essay scoring, the model is general and applicable to any scoring setting with more than one score per subject/object and recurring raters.

Our approach has several advantages over those typically used in related work. First, it provides a unified model of population-level parameters and individual raters/essays. The hierarchical structure also mitigates the problems of multiple comparisons when identifying extremely biased or unreliable raters, making the model robust to a smaller number of scores and/or raters. Finally, compared to more flexible ordinal models, we estimate the impact of rater effects on final scores directly, which substantially simplifies interpretation of rater effects and makes computation feasible. Note that the proposed model can be interpreted as an ordinal probit model with fixed equidistributed unit cut-offs.

The substantive findings based on our results agree with the prevailing empirical evidence that in essay scoring (and other similar assessments) raters are biased and unreliable to the extent of having a non-negligible impact on final scores. While most related work reports bias, only a few attempt to measure the impact of bias (and unreliability) on final scores/grades. Even in those cases, the different methodologies used make it impossible to accurately compare and consolidate the findings, so the following is approximate at best. The numbers reported in related work and reviewed in the Introduction vary, but in most cases the expected impact lies between 10% and 20% of the ratings scale. In our case, the median effect of a single rater is 3 (or 6%), which would suggest that the raters have a smaller impact. Taking into account that these are well-trained and mostly also very experienced raters, this is not surprising.

With the current two raters per essay, we estimate that about 10% of essays would be scored 5 points too high or 5 points too low only due to rater effects. This implies that 10 points gaps between essays are not uncommon, which translates into 10% of Slovenian language exam and almost 1 point on the entire Matura. If we were to correct for bias, we could mitigate some of the impact of rater effects, however, rater unreliability is a much larger source of variability and represents a vast majority of the impact on final scores. Such non-systematic error can not easily be corrected and must be managed in some other way. Excluding the most unreliable and biased raters is one option, but the gain would be small. Another option would be an additional rater. Combined with correcting for bias, however, it would only reduce the impact by approximately one third.

This study is, to the best of our knowledge, the first systematic investigation of rater effects over a period of several years. Results confirm that a rater’s bias and reliability are characteristics that persist through time, potentially with very little variability. Furthermore, we found no evidence that bias and reliability of raters as a population meaningfully change over time or with the number of essays a rater scores (a proxy for rater experience).

At the rubrics-level the overall impact of rater effects is greater in the style and structure rubrics, relative to the content and syntax rubrics. This makes sense, as it can be argued that content and syntax are better-defined and therefore easier to score. The strongest correlation is between the biases of the two bonus rubrics. These two rubrics clearly measure two distinct aspects of an essay, so this correlation can be either due to differences in overall strictness/leniency or due to the raters being unable (or unwilling, for example, giving a content bonus to additionally reward an exceptional performance in language) to distinguish between language and content when giving out bonus points. We are not able to decide between these explanations, but all of them imply misuse of the bonus points rubrics.

There are correlations in other rubric pairs as well but these are more difficult to interpret. Assuming that there is no substantial overlap between rubrics (in the sense that they measure the same aspect of an essay’s quality) then any differences in biases between different rubrics pairs would imply that the raters are not able to properly distinguish between rubrics. However, it seems more likely that observed correlations between biases are, at least partially, due to rubrics actually overlapping. The correlated rubrics do make sense: all the language rubrics are positively correlated, especially syntax and style, and not correlated with the content rubric, with the exception of content and structure, which are positively correlated.

### Directions for future work

The proposed approach could also be extended to explore several potentially interesting aspects we did not or could not investigate. One important path for future work is modelling how rater characteristics affect rater bias and reliability. For example, age, gender, and, in particular, rater experience, which would be relatively straightforward to incorporate into the model. In our study we were unable to pursue this due to lack of relevant data.

In terms of bias management, we dedicated most of our attention to the rater’s overall bias, the effect that we were primarily interested in and wanted to remove, and less to rubric-specific biases. However, a more detailed analysis of the latter, especially of whether distinct types of raters exist, would be useful in identifying particular areas of bias and unreliability and training focused on a particular area.

In this study, potential changes over time were not incorporated directly into the model. Instead, we analysed them post-hoc using the posterior distributions. Modelling all years simultaneously with a temporal component made the computation infeasible and would require a different computational approach. We did not pursue this, because we had only 5 time-points (years) of data and no other time-related information, which would allow for more certain estimates of changes over time. If short-term timestamps or data over a longer period of time were available, trends could be modelled directly.

## Supporting information

S1 Data The data used in our study. The data are stored as a serialized R programming language data.frame object (see saveRDS()). Each row represents one essay, the rater IDs and scores, and a detailed breakdown of the scores by rubric.
(ZIP)
